# Echinomycin did not affect the safety of fracture healing: an experimental pilot study on a murine femur fracture model

**DOI:** 10.1186/s13037-016-0094-9

**Published:** 2016-02-16

**Authors:** Thorsten Jentzsch, Stefan M. Zimmermann, Flora Nicholls, Paolo Cinelli, Hans-Peter Simmen, Clément M. L. Werner

**Affiliations:** Division of Trauma Surgery, Department of Surgery, University Hospital Zurich, University of Zurich, Ramistrasse 100, 8091 Zurich, Switzerland; Central Biological Laboratory, University Hospital Zurich, Ramistrasse 100, Zurich, 8091 Switzerland

**Keywords:** Heterotopic ossification (HO), Echinomycin, Bone healing, Callus formation, Murine femur fracture model

## Abstract

**Background:**

There is a need for effective drugs in the prevention and treatment of heterotopic ossifications (HO) after fractures. Echinomycin has been shown to prevent formation of HO in an animal model. However, before it may be considered as an option against HO, it needs to be studied whether it prevents fracture healing similar to non-steroidal anti-inflammatory drugs (NSAIDS). Therefore, the hypothesis was that echinomycin prevents fracture healing and callus formation.

**Methods:**

In an experimental murine pilot study, standard blunt femur fractures were induced and retrograde intramedullary compression fixation of the femur was performed. The treatment group (n = 8) received echinomycin (0.3 mg/kg body weight) and the control group (n = 8) did not receive echinomycin. The fractures and implant positions were verified by conventional X-rays immediately postoperatively. As the primary outcome variable, fracture healing (osseous consolidation) was evaluated by conventional X-rays and micro-computed tomography (CT) scans after ten weeks and graded as healed, partial or complete pseudarthrosis. The secondary outcome, callus formation, was graded semi-quantitatively from 0 (mostly absent) to 3 (maximum).

**Results:**

Fracture healing was present in all living cases after ten weeks concerning the treatment group. Partial pseudarthrosis was seen in two cases, one in the treatment and another one in the control group. Complete pseudarthrosis was seen in one case of the control group after an open fracture. Callus formation was similar in both groups with a mean grade of 1.5 within each group. Two cases of the treatment group died.

**Conclusion:**

As a novel finding, echinomycin did not inhibit fracture healing or callus formation in this in vivo murine standard femur fracture model pilot study. Further studies involving a larger number of cases, quantitative assessment with CT scans and histopathological analysis are needed before generalizing the results of this pilot study.

## Background

Heterotopic ossifications (HO) are characterized by pathologic bone formation outside of osseous tissue. The origins of HO are found in traumatic, neurogenic or genetic reasons. Traumatic etiologies involve fractures, brain injuries and burns. Thus, HO are commonly encountered by orthopedic trauma surgeons [1–3]. The exact pathogenic mechanism is not yet fully understood. Hypoxic stress may activate the deoxyribonucleic acid-(DNA-)binding activity of hypoxia-inducible factors (HIF) [4]. This may induce angiogenic stimulators, leading to the activation of vascular endothelial growth factor (VEGF) [5] and osteoprogenitor cells [6]. Vascular endothelial growth factor leads to the formation of endothelial cells and osteoprogenitor cells differentiate from mesenchymal stem cells via chondrocytes into osteoblasts. In comparison to normal callus formation, these events ultimately lead to the deposition of calcium and formation of HO [7].

Thus far, non-steroidal anti-inflammatory drugs (NSAIDS) are most commonly used drug for HO, while radiotherapy is a less commonly used option and bisphosphonates have fallen out of favor [7]. However, they do not halt the formation of HO completely, are accompanied by serious adverse effects [8, 9], and, importantly, inhibit fracture healing [10–14]. So far, surgical removal constitutes the only genuine treatment option for HO. However, the nature of an invasive procedure with its accompanied risks such as nerve lesions and, particularly, high recurrence rate remain an ongoing dilemma for doctors and patients alike. Therefore, there is an increasing need for the use of therapeutic agents in the prevention and treatment of HO [15].

Echinomycin is a cyclic peptide metabolite from Streptomyces species MST-AS5446 and acts as an antibiotic agent [16]. It belongs to the quinoxalines and has been attributed similar effects to vancomycin [17]. It is able to inhibit HIF1-alpha (α) through DNA intercalation [18]. This is important in cellular mechanisms of tumor hypoxia [19]. So far, there is only one recent study [1] that has linked echinomycin to HO. It was shown that echinomycin effectively prevents formation of HO in a murine model. This is most likely caused by interruption of inductive signaling pathways of hypoxia, HIF1-α, VEGF, and angiogenesis. If it can be demonstrated that echinomycin does not inhibit fracture healing and does not lead to an increased rate of pseudarthrosis [20–22], it may potentially become of interest for clinicians.

It remains unclear whether echinomycin can be successfully used in the prevention of HO after fracutres since there are no studies on the potential adverse effect of echinomycin on fracture healing and callus formation as seen with NSAIDS. Therefore, the hypothesis of this experimental animal pilot study was that echinomycin prevents fracture healing and callus formation.

## Methods

The experimental protocols were approved by the local institutional review committee (cantonal veterinary office) and met the guidelines of the local governmental agency.

### Animals

This study included cluster of differentiation-1 (CD1) mice because they are not genetically modified, rather large and bred locally [1]. Male mice were used because human HO formation is more pronounced in males. Mice were marked on the tail for identification. Isoflurane 2–5 % in oxygen (flow rate 400 milliliter/minute (ml/min)) via nose cone was used for anesthesia. Buprenorphine (0.05 mg/kg) was administered subcutaneously for intraoperative pain management and paracetamol (syrup 3 %, 200 milligram/kilogram (mg/kg)) was provided for 1–3 postoperative days. Mice were followed up and monitored several times a day. A score-sheet was used in order to detect any signs of distress (shivering, apathy, no water or food intake). No signs of increased distress were noted and treatment with paracetamol did not have to be extended.

### Surgery

Retrograde intramedullary compression fixation of the femur through a knee arthrotomy approach was chosen because this method allows mice to fully ambulate after surgery [23]. A five mm incision was used for a parapetallar approach. The patella was luxated laterally. A 0.5 drill bit was used for intercondylar opening of the medullary cavity. A guide wire (d = 0.2 mm) was used to assemble the needle tip (25 G force (G) x 16 mm, d = 0.55 mm) for intramedullary reaming up to the proximal femur in the area of the trochanter. The needle tip was removed while keeping the guide wire in place. Using the guide wire, the Association for the Study of Internal Fixation (AO) MouseScrew (AO foundation, Davos, Switzerland) was assembled and carefully inserted until the tip approached the area of the trochanter. Clockwise turning of the MouseScrew was initiated to secure the tip in the cortical bone. The patella was repositioned and refixated, and the wound was closed with Prolene Polypropylene Suture 7.0 (Ethicon Incorporation, Johnson and Johnson, New Brunswick, New Jersey, United States).

### Fracture model

**Fig. 1:**
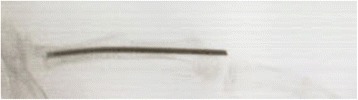
Lateral X-ray of the intramedullary compressed femur immediately postoperatively

**Fig. 2:**
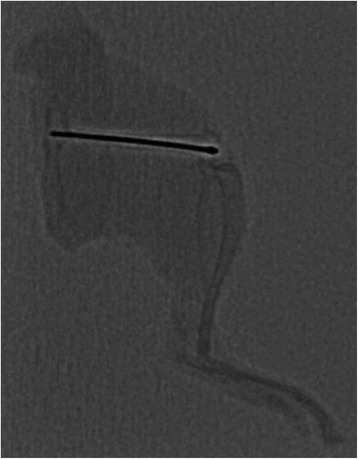
Lateral X-ray of the intramedullary compressed femur ten weeks postoperatively

**Fig. 3:**
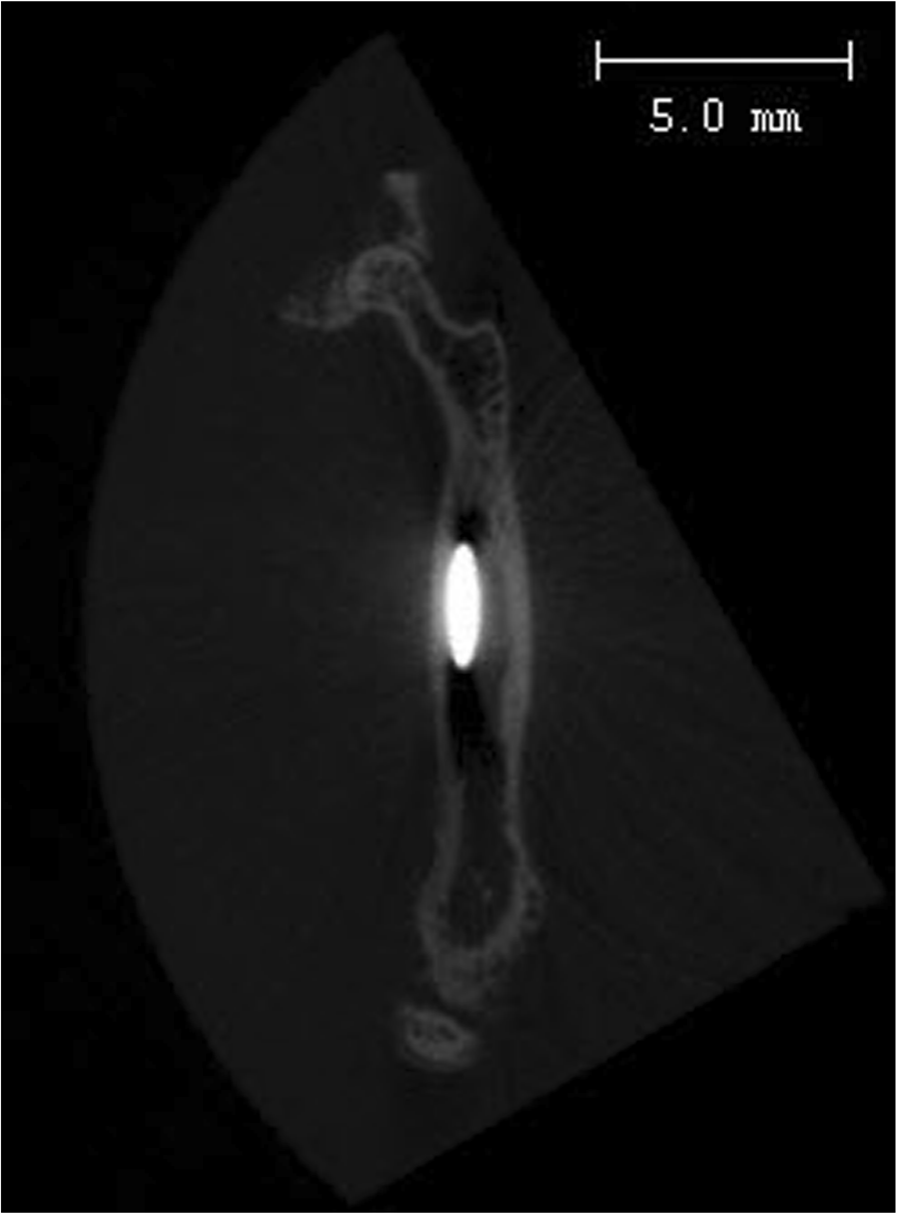
Coronal plane of a micro-computed tomography scan of the intramedullary compressed femur ten weeks postoperatively

There are open and closed standard fracture models [23–28]. Open models use osteotomies whereas closed models utilize three-point bending or drop weight. Using an established closed standard fracture model, femur fractures can be induced through a tower with a drop weight [24–26]. These fractures could be induced after performing intramedullary fixation with a pin. In mice, open osteotomies [27] or three-point fracturing devices can also be used [28]. In this murine study, a standard blunt femur fracture was induced by a fracture device (AO foundation, Davos, Switzerland) consisting of a tower with a drop weight resulting in 500 G from 35 centimeters (cm), theoretically leading to 0.5 shaft dislocation, as previously described (Figs. 1, 2, and 3) [24].

### Groups

Mice (cases) were randomly assigned to one of two groups. The treatment (echinomycin [E]) group included eight cases (n = 8, E.1 - E.8) and the control (C) group included another eight cases (n = 8, C.1 - C.8). The treatment group received echinomycin (10 microgram (mcg) [0.3 mg/kg body weight]) (Sigma-Aldrich, Buchs, Switzerland). Echinomycin was diluted in dimethyl sulfoxide (DMSO) and administered subcutaneously in the interscapular region. Injections were performed once a week for a total amount of four weeks [1]. The dosage was chosen due to the following considerations. A previous in vitro study [16] reported that the lethal dose 50 (LD50) of echinomycin was 12.3 mg/kg. However, further in vitro murine studies [29, 30] have used higher dosages of 10 mg/kg every two days for 14 days [29] and up to 40 mg/kg every two days for a total of five administrations [30] without reporting adverse effects. Since Zimmermann et al. [1] reported an effect of echinomycin on heterotopic ossification without observing any adverse or lethal effects, the same dosage was used in this study. Euthanasia and harvesting of the limbs were performed at ten weeks.

### Radiologic assessment

The fractures and implant positions were verified by conventional X-rays (Fluoroscan InSight, Hologic Incorporation, Bedford, Massachusetts, United States) immediately postoperatively (Fig. 1). After ten weeks, the harvested legs underwent a second conventional radiologic work-up (Philips Allura FD20, Koninklijke Philips Electronics Naamloze Vennootschap, Amsterdam, The Netherlands) and micro-computed tomography (CT) (nominal resolution of 30 mm, b-cube, Swiss Federal Institute of Technology, Zurich, Switzerland) with particular focus on fracture healing and callus formation (Figs. 2 and 3). The primary outcome, fracture healing was assumed with osseous consolidation of both cortices. Partial pseudarthrosis was determined as osseous consolidation of only one cortex and complete pseudarthrosis was presumed without osseous consolidation of either cortex. The secondary outcome, callus formation was graded semiquantitatively with a score from 0–3 (0 = mostly absent, 1 = minimum, 2 = medium and 3 = maximum). Radiography and micro-CTs were scored by two independent investigators. Descriptive analyses were performed (Table 1).

**Table 1:** Fracture healing, callus formation, and complications are shown for mice in the treatment (E.1 – E.8) and control (C.1 – C.8) groups

Group	Fracture healing	Callus formation (0 = mostly absent, 1 = minimum, 2 = medium, 3 = maximum)	Complications
E.1	Yes	2	Implant bending
E.2	Yes	2	No
E.3	Yes	1	Implant bending, partial pseudarthrosis
E.4	Yes	2	No
E.5	Yes	1	No
E.6	Yes	1	No
E.7	NA	NA	Died intraoperatively
E.8	NA	NA	Died one week postoperatively
C.1	Yes	1	Partial pseudarthrosis
C.2	Yes	3	No
C.3	Yes	2	No
C.4	No	0	Implant bending, open fracture, complete pseudarthrosis
C.5	Yes	2	No
C.6	Yes	1	No
C.7	Yes	1	No
C.8	Yes	2	No

## Results

### Mice

Two cases of the treatment group died. One death occurred intraoperatively, most likely due to cardiopulmonary arrest. Another death occurred about one week postoperatively due to unknown reasons. The remaining cases were able to ambulate well postoperatively. No other adverse effects were observed with administration of echinomycin.

### Fracture healing

Using a micro CT, osseous consolidation and fracture healing was observed in all (100 %) cases alive after 10 weeks in both groups (Figs. 2 and 3 and Table 1). Partial pseudarthrosis was seen in two cases: one (12.5 %) in the treatment and another one (12.5 %) in the control group. Complete pseudarthrosis was seen in one (12.5 %) case of the control group after an open fracture. The open fractures (12.5 % each) had occurred accidentally from the drop weight. Inadvertent implant bending was seen immediately postoperatively in three cases. Two (25 %) cases of the treatment group and one (12.5 %) case of the control group were affected.

### Callus formation

Callus formation was similar in both groups. The mean grade of callus formation was 1.5 within each group. Using micro-CT, some callus formation was visible in all (100 %) cases alive after 10 weeks indicating that the femoral fracture model resulted in an actual fracture in every (100 %) case.

## Discussion

This pilot study represents the first investigation about the effects of echinomycin on fracture healing and callus formation. According to the results, echinomycin does not inhibit bone healing or callus formation in a murine fracture model. This lowers the concern that echinomycin may compromise fracture healing and callus formation by blocking HIF1-α. Therefore, valuable information is added on the recently introduced idea that echinomycin may be an effective drug in the management of HO [1].

Echinomycin, an antibiotic agent inhibiting HIF1-α, has recently [1] been shown in the successful prevention of HO. In a study by Zimmermann et al. [1], the effect of echinomycin on formation of HO was examined in a murine Achilles tendon tenotomy model. Analysis with micro-CT and histology showed that echinomycin effectively reduced formation of HO in a murine model. This effect was most likely attributed to the interruption of inductive signaling pathways of hypoxia, HIF1-α, VEGF, and angiogenesis. These findings have led to the presented study because it is necessary to investigate the effects of echinomycin on fracture healing and callus formation before it can be used in the prevention of HO in a clinical setting.

The presented results add information to the current literature because the use of NSAIDS in HO has been limited due to their adverse effects and inhibition of fracture healing [10]. Adverse effects include gastrointestinal bleeding [8] and nephrotoxicity [9]. There are several studies [10–13] about the inhibition of fracture healing of NSAIDS. For example, Giannoudis et al. [13] compared 32 patients with nonunion of the femur to healed fractures and found a significant association between nonunion and the use of NSAIDS. Similarly, Burd et al. [10] reported an increased nonunion rate of 26 % in patients receiving indomethacin compared to 7 % in patients without indomethacin when looking at 282 patients with an acetabular fracture. This may be based on the fact that osseous consolidation requires an initial phase of inflammation, which is inhibited by NSAIDS [11]. More precisely, NSAIDS inhibit bone regeneration by inhibiting cyclooxygenase activity leading to a decrease in lipid mediators such as omega-(Ω-)6 fatty acids including arachidonic acid and Ω-3 fatty acids that are involved in cell signaling. The differentiation of mesenchymal cells into osteogenic cells is repressed [7]. This ultimately inhibits bone healing [12]. The presented results suggest that echinomycin may not be accompanied by these limiting factors.

Secondary bone healing with some callus formation was seen in all cases even though fractures were difficult to identify on the immediate postoperative X-rays [14]. The partial pseudarthrosis rate, which is known from humans [20], was low and one complete pseudarthrosis was most likely due to an accidental open fracture [21, 22]. It remains difficult to differentiate HO from physiologic callus formation in fractures histologically [15] and the reason for the appearance of HO without simultaneous callus formation is still debated [4, 6]. It has to be assumed that HO is formed in hypoxic soft tissue through activation of HIF and VEGF, while callus is formed around bone with normal oxygen supply. This can be exemplified by study by Rath et al. [4], who showed that HO formation could be decreased by resection of necrotic gluteus minimus muscle. In comparison to callus formation, HO also lacks periosteum [7].

The rather low dose (0.3 mg/kg body weight) of echinomycin was chosen to avoid possible adverse effects [1]. Even though two mice died, one death was probably attributed to cardiopulmonary arrest intraoperatively, while the other death remains unknown. However, in a previous human colon cancer cell line study by Park et al. [16], the lethal dose 50 (LD50) was 12.3 mg/kg body weight, which is much higher than the dose used in our study. Since Zimmermann et al. [1] did not report any adverse effects with the same dose, the cause of death is unlikely to be caused by echinomycin.

This murine standard fracture model of this study, which used a tower with a drop weight after intramedullary fixation prior to inducing the fracture [26], was able to produce fractures in all cases and provided consistent results comparable to other fracture models such as open osteotomy or closed three-point bending [31]. The drop weight resulted in implant bending in three cases and an open fracture in one case. These results add valuable information about the use of fracture models in mice [24, 26, 31]. Since the effect of echinomycin on HO has only been studied in mice, an easy-to-use standard closed femur fracture model with a drop weight was chosen in mice. In mice, potential gene defects in knockout strains may also be evaluated [32].

As a limitation, this study included a small sample size of eight cases in each group. Therefore, no power or number needed to treat analysis was performed and results need to be interpreted with care. The relatively low dose of echinomycin was chosen due to the aforementioned considerations, but may be further investigated in the future. Nonetheless, the results of the presented pilot study are pointing toward an interesting direction for potential future studies of this clinically relevant topic. Regularly spaced radiological examinations were avoided because radiation may interfere with HO [2, 3]. The use of CT scans, at least for final examinations, may be preferred because fractures are difficult to identify on conventional X-rays. These CT scans may also be used to quantitatively assess bone formation, which was not the subject of the present study. In this study, histological evaluation was not pursued. Thus, there is a potential for future studies involving a larger experimental sample size to draw statistically relevant conclusions and histological assessments in order to quantify osseous consolidation more precisely. It also seems plausible to perform experimental studies with regard to the correct dose and specific effects of echinomycin. If it was to be shown that echinomycin could be used as a regular pre- or postoperative antibiotic, it may ultimately become an option in the perioperative period in patients at risk for HO.

## Conclusion

This is the first study to investigate the effects of echinomycin on fracture healing and callus formation. Echinomycin did not seem to inhibit fracture healing or callus formation in this vivo pilot study using a murine standard femur fracture model. Further studies involving a larger sample size, quantitative assessment with CT scans and histopathological analysis may provide more generalizable information.
